# Post-exposure vaccine effectiveness and contact management in the mpox outbreak, Madrid, Spain, May to August 2022

**DOI:** 10.2807/1560-7917.ES.2023.28.24.2200883

**Published:** 2023-06-15

**Authors:** Laura Montero Morales, José Francisco Barbas del Buey, Marcos Alonso García, Noelia Cenamor Largo, Alba Nieto Juliá, María C Vázquez Torres, Susana Jiménez Bueno, Andrés Aragón Peña, Elisa Gil Montalbán, Jesús Íñigo Martínez, María Alonso Colón, Araceli Arce Arnáez, on behalf of Madrid Surveillance Network and Vaccination Centre of Madrid Region

**Affiliations:** 1 Directorate General of Public Health, Regional Ministry of Health of Madrid, Madrid, Spain; 2 The members of the networks are acknowledged at the end of the article.

**Keywords:** Post-Exposure Prophylaxis, mpox, smallpox vaccine, vaccine efficacy, contact tracing, disease outbreaks

## Abstract

**Background:**

Appropriate vaccination strategies have been key to controlling the outbreak of mpox outside endemic areas in 2022, yet few studies have provided information on mpox vaccine effectiveness (VE).

**Aim:**

To assess VE after one dose of a third-generation smallpox vaccine against mpox when given as post-exposure prophylaxis (PEP) within 14 days.

**Methods:**

A survival analysis in a prospective cohort of close contacts of laboratory-confirmed mpox cases was conducted from the beginning of the outbreak in the region of Madrid in May 2022. The study included contacts of cases in this region diagnosed between 17 May and 15 August 2022. Follow up was up to 49 days. A multivariate proportional hazard model was used to evaluate VE in the presence of confounding and interaction.

**Results:**

Information was obtained from 484 close contacts, of which 230 were vaccinated within 14 days of exposure. Of the close contacts, 57 became ill during follow-up, eight vaccinated and 49 unvaccinated. The adjusted effectiveness of the vaccine was 88.8% (95% CI: 76.0–94.7). Among sexual contacts, VE was 93.6% (95% CI: 72.1–98.5) for non-cohabitants and 88.6% (95% CI: 66.1–96.2) for cohabitants.

**Conclusion:**

Post-exposure prophylaxis of close contacts of mpox cases is an effective measure that can contribute to reducing the number of cases and eventually the symptoms of breakthrough infections. The continued use of PEP together with pre-exposure prophylaxis by vaccination and other population-targeted prevention measures are key factors in controlling an mpox outbreak.

**Key public health message**

**What did you want to address in this study?**

In 2022, when a large mpox outbreak occurred outside areas usually affected by the disease, little was known about the effect of one dose of third-generation live vaccine vaccines in a real world scenario. We wished to assess the effectiveness of vaccination against mpox, and specifically whether close contacts of confirmed mpox cases have a lower risk of getting the disease if they are vaccinated vs if they are not.

**What have we learnt from this study?**

Vaccination against mpox virus seems to be an effective measure for up to 7 weeks after vaccination. Vaccinated contacts had a lower risk of developing the disease than unvaccinated contacts and, additionally, if they got the disease, they had fewer symptoms.

**What are the implications of your findings for public health?**

Mpox post-exposure prophylaxis vaccination can contribute to the controlling of outbreaks. It is important to encourage the identification of close contacts as well as to promote vaccination so that they can benefit from reduced risk of the disease or its severity.

## Introduction

Since detection of mpox (formerly monkeypox) virus transmission outside endemic areas in early May 2022, a large multi-country mpox outbreak spread worldwide, with a total of 86,724 laboratory confirmed cases by 27 March 2023 [1]. On 23 July 2022, the World Health Organization (WHO) Director General declared this outbreak a public health emergency of international concern (PHEIC) and issued a set of temporary recommendations [2]. Until the end of July 2022, Europe remained the epicentre of this large and geographically widespread outbreak, with a steady increase of cases and affected countries [1,3]. Spain was the European country reporting most cases and the third worldwide. The capital Madrid region (6,751,251 inhabitants) [4] had the highest mpox rates in Spain with 373 cases per million inhabitants [5,6].

Both post-exposure prophylaxis (PEP) and pre-exposure prophylaxis (PrEP) by vaccination have been included among the main measures to contain the 2022 outbreak and their eligibility criteria have been included in the guidelines of both national and international organisations [7-10]. For these interventions, Modified Vaccinia Ankara – Bavarian Nordic (MVA-BN), a third-generation live vaccine authorised in 2013 by the European Medicines Agency (EMA) for the prevention of smallpox was used. Taking these recommendations into account, scientists have been trying to launch studies of vaccine efficacy and effectiveness but there are ethical, methodological and logistical challenges that are complicating the design of these studies. Consequently, robust data on the clinical efficacy or effectiveness of the third-generation vaccines against mpox disease have been lacking at the time of writing [11,12].

The aim of this observational study was to assess post-exposure vaccine effectiveness (VE) under the conditions of routine clinical practice during the early stages of the 2022 outbreak and the vaccine’s effectiveness in preventing or minimising the disease in close contacts.

## Methods

### Study design and definition of close contacts

A dynamic prospective cohort study to assess VE of one vaccine dose given as PEP in close contacts of laboratory-confirmed cases (by specific or generic Orthopoxvirus PCR) was conducted from 17 May to 15 August 2022, starting shortly after the beginning of the mpox outbreak in Madrid in early May 2022.

This study included close contacts that met the following inclusion criteria: having contact with body fluids or lesions, contaminated fomites or clinical samples (without personal protective equipment) from a case since the time of appearance of the first symptoms, or in case of presenting with a rash since the day before first symptoms appeared [13], and whose index case was laboratory confirmed infection with mpox virus. Close contacts were excluded if: the index case could not be identified, the index case could not be laboratory-confirmed, the contact had compatible symptoms with mpox and the aetiology could not be confirmed, the date of onset of risk of the contact could not be established or they had other risk exposures during the follow-up period outside their home.

The maximum follow-up period was 49 days for household members (a maximum of 28 days of symptomatic disease plus a maximum of 21 days of incubation period [13]) and 21 days for non-cohabitants.

### Contact identification and post-exposure prophylaxis

Post-exposure prophylaxis was made available for close contacts based on the criteria agreed at national level on 9 June 2022 [10] and its subsequent update [14]. Vaccination priority was given to contacts at risk of severe disease or with high risk of exposure: immunocompromised persons, pregnant women, children of any age and healthcare or laboratory personnel affected by accidental exposure. If vaccines were still available, these were then offered to contacts with high risk of exposure: sexual partners or household members/cohabitants with no possibility of self-isolation. Those with a history of smallpox vaccination were not excluded from PEP. The vaccine was offered up to 14 days post exposure, preferentially in the first 4 days. If the risk was sustained this period could be extended. Vaccination as PEP was launched on 16 June 2022.

The third-generation smallpox vaccines IMVANEX or JYNNEOS (Bavarian Nordic) were delivered subcutaneously with two 0.5 mL doses administered 28 days apart [15,16]. The administration of the second dose, depending on vaccine availability, had not yet been implemented during the study period.

Confirmed cases were asked by a trained epidemiologist about the number of close contacts and the contacts’ identifying data. The risk of each contact was assessed individually and vaccination was offered if they met the aforementioned criteria. Post-exposure prophylaxis was administered at a single centre in Madrid. At the vaccination centre, information about the vaccine and the disease was provided and an individual assessment of possible side effects was made. If the contact agreed to the vaccination, they signed an informed consent. Close contacts who received one dose of the vaccine were considered post-exposure vaccinated. Those who were contacts before the launch of PEP, those who declined vaccination, those who did not attend the scheduled appointment even if they accepted the vaccination or those who had contraindications to the vaccine were considered unvaccinated. Vaccination status was confirmed by consulting the vaccination registry. The variables collected from the contacts were: sex (legal sex as recorded in the sanitary registry), age, type of contact, use of HIV-PrEP [17] (last dispensation in the past 3 months), HIV status, date of symptom onset of index case, date of last contact with index case, vaccination date, date of symptom onset for contacts who became infected, symptoms (exanthema, lymphadenopathy, proctitis, mouth ulcers, ocular involvement, bacterial superinfection of lesions, bronchopneumonia and general symptoms such as fever, asthenia, odynophagia, muscle pain, headache, other), polysymptomatology (four or more symptoms described above) and type of confirmation test (specific or generic Orthopoxvirus PCR).

### Data analysis

Due to the lack of individualised computerised records of smallpox vaccination, a sensitivity analysis was used to assess whether close contacts had previously been vaccination against smallpox. Here, in addition to using age as a continuous variable, a dichotomous vicarious variable (*I_v_*) and its interaction with post-exposure vaccination was introduced. The cut-off point used was age since it was possible to differentiate by age between those who had or had not received a smallpox vaccination. In Spain, smallpox vaccination was mandatory until 26 October 1979 [18]. Therefore, people aged 42.2 years or older in 2022 were likely to have been vaccinated. Given that 51.8% of the confirmed cases were born in Spain and the remaining cases in South and Central American countries where the Pan American Health Organisation [19] specified that “*most people aged 50 or over would have been vaccinated against smallpox*”, the weighted cut-off point was set at 46.2 years.

### Statistical analysis

The descriptive analysis of qualitative variables was performed with frequency distribution and the quantitative variables with mean and standard deviation (SD) or median and interquartile range (IQR). To compare characteristics of the contacts (vaccinated and unvaccinated) a chi-squared test, Fisher's exact test or t-test were used depending on the type of variable (categorical or continuous). Log-rank test was used to evaluate the trends. In all analyses, a significance level of 0.05 was used.

Overall VE and its 95% confidence interval (CI) was assessed using a survival analysis between those not vaccinated and those vaccinated with PEP, with the development of an mpox infection as the main outcome.

To measure crude effectiveness of the vaccine, we performed a univariate analysis of the different characteristics of the groups, calculating the rate ratio using the Mantel-Cox method. We applied the Cox proportional hazards model to a multivariate analysis to measure adjusted VE, adjusting for the different covariates and evaluating the presence of effect modifiers. The estimation of VE was given by *VE *= (1-*HR*)×100, where HR is the hazard ratio. The detailed description of the method is available in the Supplementary material. The presence of general symptoms or polysymptomatology were the events used in the VE analysis for preventing or minimising the disease in close contacts. Uncensored observations were defined as close contacts who developed the main outcome during follow-up, while censored observations were defined as close contacts free of the main outcome at the end of the follow-up.

To assess the possible incidence bias that would cause cases with early onset not to be vaccinated and become part of the cohort of unvaccinated cases, overestimating VE, a sensitivity analysis by intention to treat was performed. Here, the estimated VE was compared with VE obtained by including the cohort of vaccinated patients who accepted vaccination but were not vaccinated. A debug of the database was carried out in order to find, study and correct lost, illicit and improbable values, as well as inconsistencies between them, especially for the variables referring to dates. The software used for data cleansing were the Pandas, NumPy and Datetime libraries in Python version 3.9.13 and for all statistical analysis Stata version 16.1 (StataCorp, Texas, United States (US)) was used.

## Results

Since the mpox outbreak began in May 2022, until 15 August 2022 2,220 cases were reported in Madrid, of which 1,384 (62.3%) were declared close contacts. In total 4,097 close contacts were recorded, 438 of which (10.7%) had identification data. Another 53 close contacts of confirmed cases were self-reported or notified by other regions. Altogether, 491 close contacts were identified for follow-up, with 400 (81.5%) detected when PEP was available on 16 June 2022. Vaccination was offered to 274 close contacts and the first PEP dose was finally administered to 233 contacts – 58.3% of the total number of identified contacts and 85.0% of the total number of contacts who were offered the vaccine (Figure and Table 1).

**Figure:**
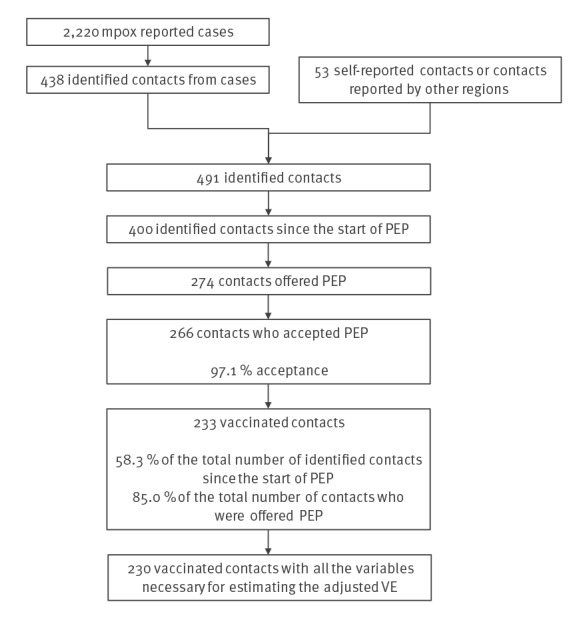
Flowchart of mpox case close contact identification and vaccination as post-exposure prophylaxis, Madrid, Spain, May–August 2022

PEP: post-exposure prophylaxis.

**Table 1:** Identification of close contacts of mpox cases and vaccination as post-exposure prophylaxis by contact type, Madrid, Spain, May–August 2022

Contact type	Sexual partners (whether or not cohabitants)		Household members (non-sexual relations)		Healthcare workers		Others (Social and nosocomial)	
	n	%	n	%	n	%	n	%
Identified contacts (n = 491)	256	52.1	187	38.1	22	4.5	26	5.3
Identified contacts since start of PEP (n = 400)	221	55.2	136	34.0	21	5.3	22	5.5
Contacts offered PEP (n = 274)	171	62.4	73	26.6	21	7.7	9	3.3
Contacts who accepted PEP (n = 266)	168	63.2	68	25.6	21	7.9	9	3.4
% Acceptance (accepted / offered)	98.2		93.2		100		100	
Vaccinated contacts (n = 233)	148	63.5	56	24.0	20	8.6	9	3.9
% Vaccinated contacts since start of PEP	67.0		41.2		95.2		40.9	
% Vaccinated contacts among offered PEP	86.5		76.7		95.2		100	

PEP: post-exposure prophylaxis.

### Vaccine effectiveness

Of the 491 contacts, there were only seven missing values (1.4%), all related to age. Three of the contacts with missing values were in the vaccinated group. Of the remaining 484 contacts (98.6%) who had information in all the variables necessary for the estimation of the adjusted VE, 10 (2.1%) were lost during follow-up.

Of these 484 contacts, 57 (11.8%) developed mpox during the follow-up. Of the 230 vaccinated, eight had a breakthrough infection (3.5%) while 49 of the 254 unvaccinated contacts developed an infection (19.3%). A comparison of the socio-demographic and clinical characteristics of these cases is shown in Table 2.

**Table 2:** Sociodemographic and clinical characteristics of close contacts of mpox cases who developed mpox during follow-up, Madrid, Spain, May–August 2022 (n = 57)

Characteristics	Cases in vaccinated individuals (n = 8) n	Cases in unvaccinated individuals (n = 49) n
Age in years (median (IQR))	35 (31–40)	34 (30–38)
Male	7	45
MSM	7	40
Born in Spain	5	24
Symptoms		
Exanthema	8	46
Lymphadenopathy	4	29
Proctitis	1	8
General symptoms	6	40
Fever	1	28
Asthenia	2	27
Odynophagia	0	16
Muscle pain	2	18
Headache	1	13
Others	2	17
Contact type		
Health worker	0	0
Nosocomial	0	0
Social contact	0	3
Household members	2	5
Cohabiting sexual	4	20
Non-cohabiting sexual	2	21
HIV-related information		
HIV-PrEP user	0	10
HIV infection	4	21
Hospitalised	0	1^a^

IQR: interquartile range; MSM: men who have sex with men; PrEP: pre-exposure prophylaxis.

^a^ Voluntarily discharged after 1 day of hospitalisation for proctitis and pain.

The total follow-up time was 11,873 days with a median of 25 days and a range of 2–46 days for censored contacts. Of the contacts, 97.7% were followed for 21 days or more. The median number of follow-up days among the 57 contacts who became ill was 8 days (range: 1–26 days).

Table 3 describes the characteristics of the contacts according to their vaccination status. The extreme age group, the type of contact and sex presented statistically significant differences according to vaccination status.

**Table 3:** Close contacts of mpox cases included in the study of vaccine effectiveness according to their vaccination status, Madrid, Spain, May–August 2022 (n = 484)

Characteristics	Contacts included in the study of vaccine effectiveness					
	Unvaccinated		Vaccinated		Total	p value
	n	%	n	%		
Total	254	52.5	230	47.5	484	0.296
Follow-up time mean (SD)						
Censored observations	26.3 (6.8)		27.0 (7.0)		26.6 (6.9)	0.286
Uncensored observations	8.0 (5.4)		13.8 (2.6)		8.8 (5.4)	0.0001
Age group in years						
0–19	12	NA	4	NA	16	0.077
20–39	135	50.2	134	49.8	269	1.000
40–59	76	47.8	83	52.2	159	0.634
60–93	31	NA	9	NA	40	0.001
Sex						
Female	78	68.4	36	31.6	114	< 0.001^a^
Male	183	48.5	194	51.5	377	
Contact type						
Health worker	2	NA	20	NA*	22*	< 0.001^b^
Nosocomial	1	NA	0	NA*	1*	
Social contact	16	NA	9	NA*	25*	
Household members	131	70.0	56	30.0	187	
Cohabiting sexual	55	40.1	82	59.9	137	
Non-cohabiting sexual	56	47.1	63	52.9	119	
HIV-related information						
HIV-PrEP user	24	NA	27	NA*	51*	0.377^a^
HIV infection	38	51.3	36	48.7	74	0.801^a^

NA: not applicable (subpopulation with small size); PrEP: pre-exposure prophylaxis.

^a^ Fisher’s exact, Table 2x2.

^b^ Fisher’s exact, Table 6x2.

Contact type was associated with becoming ill, presenting differences in survival functions of the Log-rank test (*χ^2^*(5) = 26.97, *p *< 0.001) and the trend test (*χ^2^*(1) = 17.61, *p *< 0.001). In sexual contacts (cohabiting and non-cohabiting), the observed number of cases who became ill were 67.9%* higher (47 vs 28) than expected (having the same survival function as that of total contacts). On the other hand, non-sexual household member contacts were 69.5% less likely to become ill than expected (7 vs 23). We found a similar situation according to sex that showed statistically significant differences in survival functions (*χ^2^*(1) = 7.33, *p* = 0.007) where women presented 64.3%* fewer cases than expected (5 vs 14). In those contacts using HIV-PrEP, differences in survival functions were observed (*χ^2^*(1) = 3.99, *p* = 0.046), showing a 97.3% higher risk of becoming ill than non-users. Contacts infected with HIV also presented differences in survival functions (*χ^2^*(1) = 45.47, *p* < 0.001), with a 4.02 times higher risk of becoming ill compared to those not infected.

The results of crude (rate ratio) and adjusted VE are shown in Table 4. Some CIs could not be calculated because there were no cases in that subpopulation or in the cohort of vaccinated patients.

**Table 4:** Univariate and multivariate analysis of mpox vaccine effectiveness (one dose) in close contacts of mpox cases, Madrid, Spain, May–August 2022

Variable	Vaccine effectiveness (n = 484)					
	Person-days	Events	Crude %^a^	95% CI^a^	Adjusted %	95% CI
Overall vaccine effectiveness						
Unvaccinated	5,774	49	Reference		Reference	
Vaccinated	6,099	8	84.4	66.4 to 92.8	88.8	76.0 to 94.7
Time from exposure to vaccination						
Unvaccinated	5,774	49	Reference		Reference	
0–6 days	1,152	2	81.1	20.6 to 95.5	85.5	39.3 to 96.6
7–13 days	3,233	4	85.7	59.6 to 95.0	90.2	72.5 to 96.5
14–20 days	1,595	2	82.0	24.6 to 95.7	86.7	44.0 to 96.9
21–25 days	119	0	100	NA^b^	100	NA^c^
Vaccination effectiveness by clinical symptoms						
*General symptoms*						
Unvaccinated	390	38	Reference		Reference	
Vaccinated	110	5	68.8	-1.5 to 90.4	71.6	18.1 to 90.2
*Polysymptomatic disease*						
Unvaccinated	390	29	Reference		Reference	
Vaccinated	110	2	87.5	6.2 to 98.3	85.5	26.7 to 97.1
Age in years^d^						
0–39	6,841	45	83.9	62.8 to 93.0	87.0	70.4 to 94.3
≥ 40	5,032	12	90.3	22.7 to 98.8	94.6	57.7 to 99.3
Sex						
Female	2,941	5	49.3	NA^e^	42.1	NA^e^
Male	8,932	52	88.3	73.2 to 94.9	89.5	76.4 to 95.3
Contact type						
Health worker	611	0	NA^f^	NA^f^	NA^f^	NA^f^
Nosocomial	21	0	NA^f^	NA^f^	NA^f^	NA^f^
Social contact	564	3	100	NA^b^	100	NA^b^
Household members	5,078	7	14.8	NA^e^	22.2	NA^e^
Cohabiting sexual	3,274	24	90.8	70.6 to 97.1	88.6	66.1 to 96.2
Non-cohabitant sexual	2,325	23	94.2	72.9 to 98.8	93.6	72.1 to 98.5
HIV-related information						
*HIV-PrEP user*						
Yes	1,139	10	100	NA^c^	100	NA^c^
No	10,734	47	79.4	55.1 to 90.5	85.0	67.4 to 93.1
*HIV infection*						
Yes	1,564	25	88.7	61.8 to 96.7	86.8	60.2 to 95.6
No	10,309	32	85.5	58.1 to 95.0	90.9	73.6 to 96.9

CI: confidence interval; NA: Not applicable; PrEP: pre-exposure prophylaxis.

^a^ Rate ratios using the Mantel-Cox method (stratified by time).

^b^ No cases in vaccinated group.

^c^ p value = 1.

^d^ The age subgroups of 0–20 years and 60 or more years have been merged with their neighbouring groups due to having few cases.

^e^ CI not meaningful.

^f^ No cases for this subpopulation.

The effectiveness of PEP, controlling for type of close contact, sex, age, HIV infection and HIV-PrEP use, is 88.8% (95% CI: 76.0–94.7) in preventing the development of the disease in close contacts of mpox cases, or equivalently, unvaccinated close contacts of mpox cases post-exposure have an 8.9 times higher risk of developing the disease than vaccinated close contacts (95% CI: 4.2–19.1).

For close contacts who developed the disease after having received post-exposure vaccination, VE in reducing general symptomatology (i.e. the presence of at least one of the following symptoms: fever, asthenia, arthromyalgia, odynophagia and headache) was 71.6% (95% CI: 18.1–90.2) and 85.5% (95% CI: 26.7–97.1) in avoiding polysymptomatic disease. The different VEs (Table 4) (based on the time from exposure to vaccination) are similar to the overall VE.

For close sexual contacts, the adjusted estimated point of VE for non-cohabitants was slightly higher than for cohabitants. This result was also seen among HIV-infected cases vs non-HIV infected cases. As for the influence of age, a 10-year increase in age reduced the risk of contracting the disease among close contacts by 26.8% (95% CI: 3.2–44.6).

The interaction of the proxy smallpox vaccination variable (*I_v_*) with the study factor was not statistically significant from the likelihood ratio (*χ^2^*(3) = 2.32, *p* = 0.509). Furthermore, age did not modify the effect of PEP as its interaction was not statistically significant (*χ^2^*(1) = -13.73, *p* = 1). Additionally, the VE obtained in the subpopulation that would not have received smallpox vaccination in childhood (87.0, 95% CI: 70.4-94.3)* is similar to that estimated in the present study (88.8%, 95% CI: 76.0–94.7). The previously mentioned interaction was not statistically significant in the models for the assessment of VE in the reduction of general symptoms (* χ^2^*(1) = 1.33, *p* = 0.248) nor in polysymptomatic affectation (*χ^2^*(1) = -0.11, *p* = 0.742).

For the sensitivity analysis to assess possible incidence bias, 37 cases were included in the cohort of vaccinated patients who accepted vaccination but were not vaccinated, of which six fell ill (on 3, 7, 7, 8, 11 and 12 days post-exposure) before the date of their vaccination appointment. The VE by intention to treat was 85.4% (95% CI: 72.6–92.2), thus not substantially different from the estimated VE, which was 88.8% (95% CI: 76.0–94.7) without this factor. The vaccination delayed onset of symptoms by 5.5 days (95% CI: 1.7–9.4) although a similar delay was seen in the intention-to-treat analysis where the delay was 3.7 days (95% CI: 0.6–6.9).

## Discussion

Using regional data from Madrid during the early phase of the mpox outbreak that began in May 2022, we estimate an adjusted VE of 88.8% (95% CI: 76.0­–94.7) with one dose of the third-generation smallpox vaccine used in the context of PEP. This high effectiveness was observed in both cohabiting sexual and non-cohabiting sexual contacts and in HIV-infected contacts, although there was a slight decrease in effectiveness with respect to uninfected patients and the effectiveness was independent of the time from exposure to vaccination. The benefits of vaccination were shown in all age groups and in men. In women, given that 68.4% were not vaccinated and considering the low number of events (one in the vaccinated and four in the unvaccinated group), no conclusions can be drawn regarding VE.

We found that breakthrough infections did not exhibit severe symptoms (none required hospitalisation). Furthermore, there were fewer symptoms, a reduction in general symptoms of at least 18.1% and a reduction in polysymptomatic disease of at least 26.7% in vaccinated cases compared with unvaccinated cases. This has also been described for other infectious diseases, in which post-exposure vaccination can modify the course of the disease and reduce its severity [20].

Robust data on the clinical VE against mpox are still lacking, but some initial assessments are available, with information mainly from observational studies. Most of the published studies on VE in the 2022 mpox outbreak were carried out in the context of pre-exposure vaccination, with third-generation vaccines and a single dose. A study carried out in England [21] showed VE against symptomatic mpox of 78% (95% CI: 54–89) with a range of 71–85 in the sensitivity analysis. Another study conducted in Israel estimated an adjusted VE of 86% (95% CI: 59–95) [22]. In the US, it was observed that the incidence rates were 14 times higher among unvaccinated men (mixed PEP and PrEP), while in our current study, they were 8.9 times higher in the exclusive context of PEP for people of both sexes [23].

Two observational studies conducted in France have been published, one in Paris with 276 participants [24] and another in Lyon with 108 participants [25], in which the effect of post-exposure vaccination against mpox has been evaluated with a single dose of a third-generation smallpox vaccine. Vaccine effectiveness results were not provided, but 4% and 10% of infections in the Paris and Lyon studies, respectively, were identified after vaccination. The US study [23] identified 2.4% of all infections as breakthrough infections, a similar result to ours, which was 3.5%.

Our study has several limitations derived from its observational design. The VE found may not be generalisable because the population offered the vaccine was prioritised among the highest risk population. Although this limitation may exist for the general population because of different exposure patterns, it does not exist for the target population in the early outbreak. Furthermore, because this population was prioritised for ethical reasons, given the a priori vaccine efficacy and because of availability and high acceptance of vaccination, immunisation was not randomised. Therefore, the non-vaccinated cohort includes close contacts exposed just prior to the start of the vaccination campaign, as a regression discontinuity design would.

The possible non-detection of cases is deemed minimal, with access to emergency medical services or sexually transmitted infection (STI) clinics being free of charge and irrespective of residency status in the community and clinical laboratories in the region reporting mpox-specific test results electronically. Linkage of test results from contacts who progressed to cases depends on identification.

Selection bias may have occurred due to the difficulty in including all close contacts because they were unknown to the index case (group sex, sauna attendance, cruising etc.). Therefore, it was not possible to assess the characteristics of these contacts or determine whether they differed from the analysed contacts. Also, with the possibility of subclinical diagnoses of the close contacts most exposed to risk or contacts who were periodically monitored, a detection bias could have occurred. There could have also been a response bias between those who accepted vaccination and those who declined it, although this would be negligible given vaccination acceptance was 97.1%.

A possible limitation is that it was not possible to carry out a sensitivity analysis with gender because it was not included in the sanitary registry. The register records only binary legal sex.

Another potential limitation would be the possible existence of an incidence bias that would cause contacts with early disease onset to be in the unvaccinated cohort because they failed to arrive in time for vaccination, which would lead to an overestimation of VE. To assess the possible magnitude of such an incidence bias, an intention-to-treat sensitivity analysis was performed, which showed little change in VE (85.4% for intention-to-treat vs 88.8% for estimated VE). Another impact of this bias would be the delay in onset of symptoms in vaccinated patients compared with unvaccinated patients. The mean delay in the vaccinated cohort was 5.5 days (95% CI: 1.7–9.4) and the mean delay in the intention to vaccinate cohort was 3.7 days (95% CI: 0.6–6.9). Since the delay was observed in both groups, this difference could be due to the fact that the mildness of the symptoms and the history of vaccination delayed the diagnosis.

Another limitation is the VE results in different subpopulations with small simple sizes and wide confidence intervals. These should be interpreted with caution and the lower limit of the confidence intervals taken as the most reliable measure.

The proxy smallpox vaccination variable did not modify the effect of PEP vaccination. Age did not modify the effect either and VE in the age group that did not receive the smallpox vaccine (87.0%) was similar to the age group that did (88.8%). This suggests that prior smallpox vaccination would not modify the post-exposure effect of vaccination against mpox.

Calendar time was not included in the analysis as all participants were exposed to a laboratory-confirmed mpox case at the time of inclusion in the study, therefore the data did not depend on the changing incidence of cases over time. Likewise, diagnostic methods and clinical management did not vary throughout the study, given the relatively short duration of this study, which was sufficient to detect new incident cases based on the incubation period described for the disease and short enough to avoid repeated risk exposures outside the cohabitation setting.

The data collected by the trained epidemiologist would largely avoid any observer or interviewer bias and the review of clinical histories and microbiological results would avoid any possible recall bias.

Among the WHO recommendations to reduce the spread of mpox is the collection of data from sexual partners of confirmed cases to facilitate disease detection. The WHO also propose actions to improve notification of close contacts [26]. When following these recommendations, our main challenges were the identification and vaccination of contacts within the first 4 days after exposure. From the moment the index case sought medical care, the time elapsed between confirmation of the diagnosis, notification of the case, conduction of the epidemiological survey, communication with contacts, assessment of risk to offer PEP and making an appointment for the vaccination exceeds 4 days in a high percentage of cases. This makes it difficult to achieve the objective of vaccinating close contacts within 4 days in order to prevent the infection. Given the high VE of PEP, the delay in recruiting contacts and the fact that half of contacts were sexual partners, many of them could have benefited from PrEP without the need for a confirmed case in their personal environment. Our results show a higher acceptability of PEP (97.1%) than a recent online survey conducted in the WHO European Region (82%) [27]. In our study, 86.5% of sexual contacts who were offered the vaccine became vaccinated while the percentage was ca 10% lower for non-sexual household contacts, possibly reflecting the higher risk perceptions in the former group.

Targeted vaccination strategies, along with other measures aimed at reducing risk behaviours [28], risk communication and community engagement, early diagnosis, isolation and effective contact tracing are suggested to have been key to controlling the 2022 mpox outbreak [12].

## Conclusions

Post-exposure vaccination of close contacts of mpox cases is an effective measure that can contribute to controlling the spread of the disease and eventually the symptoms of breakthrough infections. The continued use of PEP vaccination together with PrEP and all other population-targeted prevention measures are key actions in controlling large mpox outbreaks.

## *Author’s correction

At the request of the authors, we corrected several entries in Table 3: In the first three rows of the block on contact type and in the first row of the block on HIV-related information, the vaccinated percentages have been removed and the row totals added. In addition, the following numbers were corrected in the Results chapter ‘Vaccine effectiveness’: 66.0% was changed to 67.9%; 63.6% was changed to 64.3%; (86.8%, 95% CI: 69.8–94.2) was changed to (87.0, 95% CI: 70.4-94.3). These changes were made at the request of the authors on 22 June 2023 and are marked with asterisks.

## Supplementary Data

383000 Supplementary Material
