# Author’s correction for Euro Surveill. 2023;28(24)

**DOI:** 10.2807/1560-7917.ES.2023.28.25.230622c

**Published:** 2023-06-22

**Authors:** Eurosurveillance editorial team

**Affiliations:** 1 European Centre for Disease Prevention and Control (ECDC), Stockholm, Sweden

At the request of the authors, we corrected several entries in Table 3 of the article entitled ‘Post-exposure vaccine effectiveness and contact management in the mpox outbreak, Madrid, Spain, May to August 2022’ by Montero Morales et al.: In the first three rows of the block on contact type and in the first row of the block on HIV-related information, the vaccinated percentages have been removed and the row totals added. In addition, the following numbers were corrected in the Results chapter ‘Vaccine effectiveness’: 66.0% was changed to 67.9%; 63.6% was changed to 64.3%; (86.8%, 95% CI: 69.8–94.2) was changed to (87.0, 95% CI: 70.4-94.3). These changes were made on 22 June 2023 and are marked with asterisks. 

