# Supplementary material for: Post-exposure vaccine effectiveness and contact management in the mpox outbreak, Madrid, Spain, May to August 2022
Source: Euro Surveill. 2023 Jun 15;28(24):2200883. doi: 10.2807/1560-7917.ES.2023.28.24.2200883 (PMC10318941; doi:10.2807/1560-7917.ES.2023.28.24.2200883)
Supplement: Supplementary Material [file 22-00883_GARCIA_Supplement.pdf]

POST-EXPOSURE VACCINE EFFECTIVENESS AND CONTACT MANAGEMENT IN THE MPOX OUTBREAK, MADRID, SPAIN, MAY TO AUGUST 2022

MODELING AND DATA ANALYSIS

This supplementary material is hosted by Eurosurveillance *as supporting information alongside the article* "Post-exposure vaccine effectiveness and contact management in the mpox outbreak, Madrid, Spain, May to August 2022", *on behalf of the authors, who remain responsible for the accuracy and appropriateness of the content. The same standards for ethics, copyright, attributions and permissions as for the article apply. Supplements are not edited by* Eurosurveillance *and the journal is not responsible for the maintenance of any links or email addresses provided therein.*

Analysis plan

For measurements of crude effectiveness (univariate analysis), the Mantel-Cox method was used to estimate rate ratios [1].

For measuring the adjusted vaccine effectiveness, a multivariate proportional hazards model [2] was constructed to evaluate the effect in the presence of confounding and interaction. In this model the instantaneous hazard function or instantaneous failure rate at the time of analysis t for the covariate vector $\mathbf{x} = (x_1, x_2, x_3, \ldots, x_m)^\mathsf{T}$ is given by:

$$h(t, \mathbf{x}) = h_0(t) \cdot \mathrm{e}^{\boldsymbol{\beta}^\mathsf{T}\mathbf{x}}$$

where $h_0(t)$ is the baseline hazard function at instant t and $\boldsymbol{\beta}^\mathsf{T} = (\beta_1, \beta_2, \beta_3, \ldots, \beta_m)$ denotes the transpose of the coefficient vector $\boldsymbol{\beta}$, where only $h_0(t)$ depends on time, the hazard ratio ($HR_{j/i}$) of the mode of the values of the variables $\mathbf{x}_j$ with respect to that of the values $\mathbf{x}_i$:

$$\begin{aligned} HR_{j/i} &= \frac{h(t_0, \mathbf{x}_j)}{h(t_0, \mathbf{x}_i)} = \frac{h(t_1, \mathbf{x}_j)}{h(t_1, \mathbf{x}_i)} \\ &= \cdots = \frac{h(t_n, \mathbf{x}_j)}{h(t_n, \mathbf{x}_i)} = \cdots = \mathrm{e}^{\boldsymbol{\beta}^\mathsf{T}(\mathbf{x}_j - \mathbf{x}_i)} \end{aligned}$$

being therefore constant and independent of time. Accordingly, the model assumes that the effect of the different independent variables is constant over time.

For model fitting [3, 4], the starting point was a hierarchical model that included the first-order interactions of the study factor (vaccination status) with the other control covariates (age, sex, type of close contact, HIV-PrEP user and HIV infection) and their interactions with each other. The model coefficients ($\boldsymbol{\beta}$) were estimated using the exact partial-likelihood method to handle tied failures and Breslow's partial-likelihood method to obtain the Schoenfeld residuals and scaled Schoenfeld residuals because they were not available with the previous method. In the fitting process, interactions that were not statistically significant – $p > 0.05$ in the likelihood ratio test (LR-test) - were eliminated in inverse order of significance, after which the elimination of the possible confounding factors of the model was assessed – also in inverse order of statistical significance – and they were eliminated if the variation in the estimation of the HR ($\widehat{HR}$) of the study factor was less than 5% and the accuracy of the estimation (variation in the width of the confidence interval of less than 5%) was not impaired.

After fitting the multivariate model, the proportional-hazards assumption, and the assumed log-linear relationship between the continuous variables – in the present case, age – were examined. To verify that the proportional-hazards assumption of the model was not violated, a log-log plot of survival, Kaplan–Meier, predicted survival plot, and Schoenfeld residuals and the assessment of the interactions of the different variables with the time of analysis using the LR-test were applied.

Residual analysis included Schoenfeld residuals, scaled Schoenfeld residuals, Cox-Snell residuals, deviance residuals and martingale residuals. Likelihood displacement values, LMAX values, and DFBETAs influence measures were used to assess possible influence values.

Sensitivity analyses were performed to assess the history of previous vaccination against smallpox (introduction of a vicarious variable I_v) and incidence bias (intention-to-treat analysis).

Additionally, to assess the possible incidence bias, an analysis of delay in the onset of symptoms in vaccinated contacts who developed the disease as compared to unvaccinated contacts, was performed.

Construction and testing of the multivariate proportional hazards model

The variables entered in the hierarchical model were: vaccination variable V_g (categorical variable coded in the article mentioned 5 categories), type of close contact C (categorical variable coded in the following categories: health worker, nosocomial, social contact, household members, cohabiting sexual and non-cohabitant sexual), age (A) in years (continuous variable); and sex (S), HIV infection pre-exposure prophylaxis (P_{EP}) and HIV infection (H) as dichotomous variables. Categorical variables with $n > 2$ categories were introduced as $n - 1$ dummy variables.

Starting from a model with 6 variables and 10 first-order interactions as follows:

$$V_g \times C,\ \ V_g \times A,\ \ V_g \times S,\ \ V_g \times P_{EP},\ \ V_g \times H,$$
$$C \times S,\ \ C \times P_{EP},\ \ C \times H,\ \ S \times P_{EP},\ \ S \times H$$

(the interaction $P_{EP} \times H$ was not included for being mutually exclusive) all of them have been eliminated, stepwise one by one, from the model for presenting all of them $p > 0.05$ in the LR-test, with the lowest p for the interaction $V_g \times H$ ($\chi^2_4 = 6.34, p = 0.175$). In the evaluation of the elimination from the model of the possible confounders (C, A, S, P_{EP} and H) the only variable eliminated from the model was the sex variable, since its exclusion from the model only caused a percentage variation in the estimation of 0.17%. The variation in the remaining variables exceeded 5%, reaching variations of 26.2% for the type of contact variable, thus leaving, in the final model, the study factor (V_g) and the variables A, C, P_{EP} and H. The overall fit of the model is statistically significant in the LR-test ($\chi^2_{11} = 111.62, p < 0.001$).

In the post-estimation analysis of the model, there were no violations of the proportional-hazards assumption with the test of proportional-hazards assumption by time of analysis ($\chi^2_{11} = 15.63, p = 0.155$) or by the Kaplan-Meier treatment of time ($\chi^2_{11} = 14.39, p = 0.212$). The assessment of the interactions of the variables with the time of analysis have all been found to be non-significant in the LR-test: V_g ($\chi^2_4 = 5.50, p = 0.24$), A ($\chi^2_1 = 0.58, p = 0.447$), C ($\chi^2_4 = 5.35, p = 0.253$), P_{EP} ($\chi^2_1 = 0.38, p = 0.540$) and H ($\chi^2_1 = -0.25, p = 0.619$); which shows that their effect does not vary over time. On the other hand, the observed Kaplan-Meier survival curves vs the curves predicted by the Cox model [5], obtained by the Stata post-estimation procedure with `stcoxkm`, show a practical overlap for all the variables (not applicable to age, for being a continuous variable), which indicates that the proportional hazards assumption has not been violated [6]; Figure 1 shows the closeness between the observed and predicted curves for the vaccination variable.

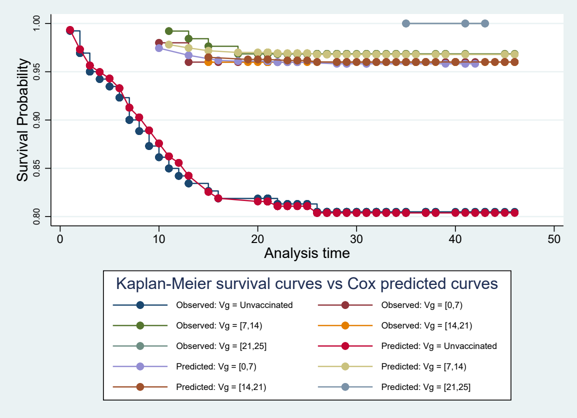

Figure 1. Kaplan-Meier survival curves vs Cox predicted curves

The analysis of the martingale residuals with respect to age (A) shows the linearity of this variable in the locally weighted regression of martingale residuals on age, thus not failing to meet the assumption of a log-linear relationship (Figure 2); while the Cox-Snell residuals follow approximately a standard censored exponential distribution with a hazard ratio of one, or equivalently, the plot of cumulative hazard for the Cox–Snell residuals against Cox-Snell residuals themselves should have approximately a slope equal to one, indicating the overall robustness of the model fit (Figure 3).

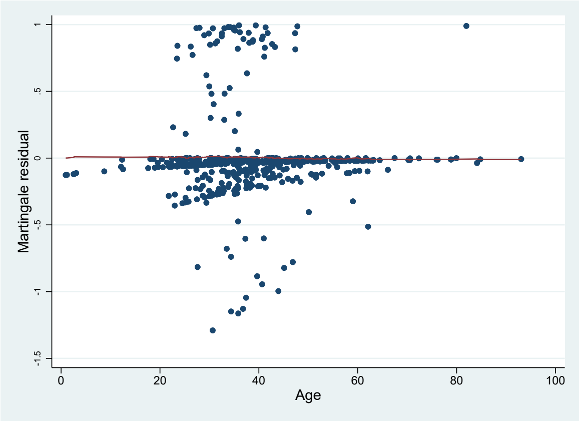

Figure 2. Plot of martingale residuals versus age and locally regression of martingale residuals on age

The influential observations detected by the measures of influence likelihood displacement values and DFBETAs correspond to the 8 cases in the vaccinated cohort who developed the disease.

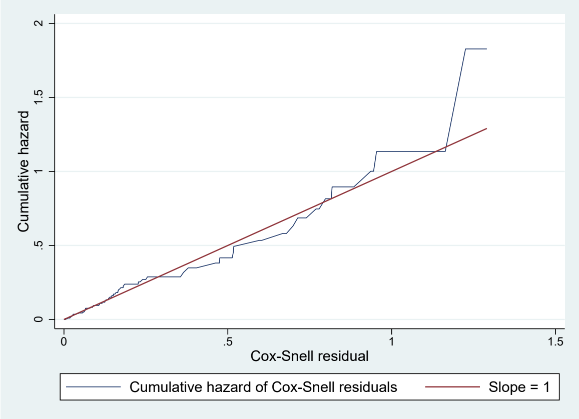

Figure 3. Goodness-of-fit plot

In the models in which the effectiveness of vaccination in reducing the symptoms of cases who developed the disease after post-exposure vaccination has been evaluated, given the small number of observations (57) which limits the introduction of independent factors, the study factor (vaccination) was introduced in the model as a dichotomous variable (V) and no effect modifications (interactions) were evaluated, except for the interaction of the proxy smallpox vaccination variable I_V with post-exposure vaccination ($I_V \times V$), which was not statistically significant ($\chi^2_1 = 1.33, p = 0.248$). Otherwise, the same criteria were followed, and the confounders were the same as

in the previous model for the model of general symptoms; in the model for polysymptomatic disease, HIV infection was not a confounder (variation of 0.8%) and the previously mentioned interaction ($I_V \times V$) was not significant either ($\chi^2_1 = 0.11, p = 0.742$). The significance of the model fit in the LR-test were ($\chi^2_5 = 15.1, p = 0.014$) and ($\chi^2_6 = 13.61, p = 0.01$) respectively for the model where the presence of general symptoms were assessed and for the polysymptomatic disease model.

For the analysis of the delay in the onset of symptoms in vaccinated patients who developed the disease, a linear regression was fitted and controlled using the same factors, eliminating from the model those that caused variations in the regression coefficient of less than 5% in both the per-protocol and intention-to-treat assessments.

References

[1] David Clayton and Michael Hills. Statistical models in epidemiology. OUP Oxford, 2013.

[2] David R Cox. Regression models and life-tables. Journal of the Royal Statistical Society: Series B (Methodological), 34(2):187–202, 1972.

[3] Raymond S Greenberg and DG Kleinbawm. Mathematical modeling strategies for the analysis of epidemiologic research. Annual Review of Public Health, 6(1):223–245, 1985.

[4] Tyler J VanderWeele. Confounding and effect modification: distribution and measure. Epidemiologic methods, 1(1):55–82, 2012.

[5] JD Kalbfleisch and RL Prentice. The survival analysis of failure time data. 2nd. New York: Wiley, 2002.

[6] Joanne Garrett et al. Predicted survival curves for the cox proportional hazards model. Stata Technical Bulletin, 8(44), 1999.
